# Association between height-related polymorphism rs17081935 and reduced handgrip strength in relation to status of atherosclerosis: a cross-sectional study

**DOI:** 10.1186/s12199-021-01000-9

**Published:** 2021-08-26

**Authors:** Yuji Shimizu, Shin-Ya Kawashiri, Kazuhiko Arima, Yuko Noguchi, Hirotomo Yamanashi, Kenichi Nobusue, Fumiaki Nonaka, Seiko Nakamichi, Yasuhiro Nagata, Takahiro Maeda

**Affiliations:** 1 Department of General Medicine, Nagasaki University Graduate School of Biomedical Sciences, Nagasaki-shi, Sakamoto 1-12-4, Nagasaki, 852-8523 Japan; 2 Department of Cardiovascular Disease Prevention, Osaka Center for Cancer and Cardiovascular Diseases Prevention, Osaka, Japan; 3 Department of Community Medicine, Nagasaki University Graduate School of Biomedical Sciences, Nagasaki, Japan; 4 Department of Public Health, Nagasaki University Graduate School of Biomedical Sciences, Nagasaki, Japan; 5 Department of Islands and Community Medicine, Nagasaki University Graduate School of Biomedical Sciences, Nagasaki, Japan

**Keywords:** Atherosclerosis, Older, Handgrip, Height, Mitochondrial metabolism, Muscle, rs17081935, SNP

## Abstract

**Background:**

Aging is a process that increases oxidative stress. Increased oxidative stress leads to the development of atherosclerosis and mitochondrial dysfunction. Mitochondria contribute to energy production that might have a beneficial influence on maintaining muscle strength. Therefore, the height-related single nucleotide polymorphism (SNP) rs17081935, which is also reported to be associated with mitochondrial metabolism, might be associated with reduced muscle strength and this association might be affected by atherosclerosis status. To clarify those associations, a cross-sectional study of 1374 elderly Japanese individuals aged 60–89 years was conducted.

**Methods:**

Logistic regression was used to clarify the association between rs17081935 and reduced handgrip strength. Since atherosclerosis might affect handgrip strength, participants were stratified by atherosclerosis status. Reduced handgrip strength was defined as being in the lowest quintile of handgrip strength (< 25.6 kg for men and < 16.1 kg for women).

**Results:**

No significant associations were found between a minor allele of rs17081935 and reduced handgrip strength among elderly participants without atherosclerosis. A significant inverse association was observed among elderly participants with atherosclerosis. After adjusting for known cardiovascular risk factors and height, the adjusted odd ratio (OR) and 95% confidence interval (CI) for reduced handgrip strength and a minor allele of rs17081935 were 1.13 (0.86, 1.43) for elderly participants without atherosclerosis and 0.55 (0.36, 0.86) for those with atherosclerosis, respectively.

**Conclusion:**

A minor allele of the height-related SNP rs17081935 was significantly inversely associated with reduced handgrip strength among older individuals with atherosclerosis, but not among those without atherosclerosis.

**Supplementary Information:**

The online version contains supplementary material available at 10.1186/s12199-021-01000-9.

## Introduction

Aging is a process that increases oxidative stress [1]. Oxidative stress causes mitochondrial dysfunction [2]. Since mitochondrial metabolism contributes to energy production that might have a beneficial effect on maintaining muscle strength, higher levels of oxidative stress might cause age-related muscle loss [3].

Genetic factors related to mitochondrial metabolism and the rs17081935 single nucleotide polymorphism (SNP), also a known height-related genetic factor [4, 5], might be associated with reduced muscle strength. Oxidative stress could affect that association.

In addition, increase levels of oxidative stress activate vascular inflammation [6] and lead to the development of atherosclerosis [7]. Therefore, analyses limited to participants with atherosclerosis could exaggerate the influence of increased oxidative stress and the strength of the beneficial influence of genetic factor on maintaining muscle strength among older individuals. We hypothesized that a beneficial association between rs17081935 and reduced muscle strength might be observed only in participants with atherosclerosis.

Increased oxidative stress decreases peripheral erythrocyte count because of reduced erythrocyte survival [8]; participants with increased oxidative stress have lower peripheral erythrocyte counts than those without increased oxidative stress. Therefore, reduced handgrip strength might be associated with lower peripheral erythrocyte count, which acts as an indicator of the influence of oxidative stress on muscle strength.

Angiogenesis is a muscle adaptation to hypoxia [9]. Angiogenesis contributes to reduced oxidative stress. Thus, the influence of decreased peripheral erythrocyte count related to oxidative stress might be observed only among participants without a beneficial genetic factor that contributes to the maintenance of muscle strength [8].

We also hypothesized that participants with atherosclerosis have significantly lower erythrocyte counts than participants without atherosclerosis who are CC-homozygotes for the rs17081935 SNP.

To test these hypotheses, we conducted a cross-sectional study of 1374 older Japanese aged 60–89 years who participated in an annual health checkup in 2017.

## Material and methods

### Study population

Methods pertaining to present risk surveys, including genetic factors, have been previously described [10–12].

This study took place in the city of Goto, which is on a remote island in western Japan. According to 2013 estimates from the National Institute of Population and Social Security Research in 2013, there were 16,264 residents aged 60–89 years in 2015 and 15,807 residents in that age group in 2020 [13].

The study population comprised 1388 individuals (501 men and 887 women) aged 60–89 years in Goto who participated in an annual health checkup in 2017. The local government conducted this annual checkup program. The program was directed by the Ministry of Health, Labour, and Welfare of Japan. Based on this annual health checkup, we performed an rs17081935-related survey to clarify the mechanism of aging, including the decrease in handgrip strength. The details of the present survey are described elsewhere [14].

Participants without serum data (*n* = 11) and SNP data (*n* = 3) were excluded from the analysis. Finally, 1374 older Japanese individuals (498 men and 876 women), with a mean age of 72.8 ± standard deviation (SD) of 7.1 years, were enrolled in the study.

### Data collection

The experimental protocols were reviewed by medical staff in meetings before the study to reduce inter-observer variability in the information collected from medical interviews and measurements. Trained interviewers obtained the medical history of the participants and information on drinking and smoking habits. Body weight and height with bare feet and while wearing light clothes were measured using an automatic body composition analyzer (BF-220; Tanita, Tokyo, Japan). Body mass index (BMI) was calculated as weight (kg)/height (m)^2^. BMI was categorized as low (< 18.0 kg/m^2^), normal (18.0–24.9 kg/m^2^), or high (≥ 25.0 kg/m^2^).

After at least 5 min of rest, systolic blood pressure (SBP) and diastolic blood pressure (DBP) were measured in the sitting position using a blood pressure measuring device (HEM-907; Omron, Kyoto, Japan). If the participants had high blood pressure measurements (SBP ≥ 140 mmHg or DBP ≥ 90 mmHg), we measured blood pressure again, and the lower blood pressure values were used. Hypertension was defined as SBP ≥ 140 mmHg, DBP ≥ 90 mmHg, or use of antihypertensive medication.

Fasting blood samples collected in siliconized tubes were used to measure low-density lipoprotein cholesterol (LDLc), high-density lipoprotein cholesterol (HDLc), and triglycerides (TG). Blood samples in sodium fluoride tubes were used to measure glycated hemoglobin (HbA1c). Measurements were obtained following standard laboratory procedures at SRL, Inc. (Tokyo, Japan). Dyslipidemia was defined as LDLc ≥ 140 mg/dL, HDLc < 40 mg/dL, TG ≥ 150 mg/dL, or lipid-lowering medication use. Diabetes was defined as HbA1 ≥ 6.5% or use of medication to lower glucose levels.

Blood samples in siliconized tubes were also used to measure creatinine. Glomerular filtration rate (GFR) was estimated using a recently adapted established method introduced by a working group of the Japanese Chronic Kidney Disease Initiative [15]: GFR (mL/min/1.73 m^2^) = 194 × (serum creatinine [enzyme method]) ^−1.094^ × (age) ^−0.287^ × (0.739 for women). Mild reduced renal function was defined as GFR of 60–89 mL/min/1.73 m^2^. Chronic kidney disease was defined as GFR < 60 mL/min/1.73 m^2^.

Samples from the EDTA-2 K tube were used to measure erythrocyte count using an automated procedure at SRL, Inc. (Tokyo, Japan).

Genomic DNA was extracted from 2 mL of whole peripheral blood using Gene Prep Star NA-480 (Kurabo Industries Ltd., Osaka, Japan). Participants were genotyped for the rs17081935 SNP using the TaqMan SNP Genotyping Assay (C_33131398, Thermo Fisher Scientific, Tokyo, Japan) and the LightCycler 480 thermal cycling platform (Roche Diagnostics, Basel, Switzerland). In detail, genomic DNA was amplified using polymerase chain reaction (PCR) (first step, 95 degrees for 30 s; second step, 40 cycles between 95 degree for 5 s and 60 degrees for 30 s; third step, 50 degrees for 30 s) with two fluorogenic hydrolysis probes (VIC/FAM). Endpoint genotyping analysis was performed. No increments in fluorescence intensity were detected during PCR from the negative control wells, which did not contain any genomic templates.

Carotid intima-media thickness (CIMT) was measured based on ultrasonography of the left and right carotid arteries by experienced vascular technicians using LOGIQ Book XP with a 10-MHz transducer (GE Healthcare, Milwaukee, WI, USA). Maximum values for left and right common carotid CIMT were calculated with semi-automated digital edge-detection software (Intimascope; MediaCross, Tokyo, Japan); the protocol is described in detail elsewhere [16]. Atherosclerosis was defined as CIMT ≥ 1.1 mm, as in our previous studies [17, 18].

Handgrip strength was determined with a handgrip dynamometer (Smedley; Matsumiya Ika Seiki Seisakujo, Tokyo, Japan) as grip strength from two measurements obtained for each hand; the maximum value for each hand was used. Reduced handgrip strength was defined as being in the lowest quintile of sex-specific handgrip strength values, < 25.6 kg for men and < 16.1 kg for women.

### Statistical analysis

The characteristics of the study patients in relation to rs17081935 genotype are expressed as means ± SD for continuous values (age and height) and percentages for prevalence data. Significant differences involving the rs17081935 genotype were evaluated using analysis of variance.

By using analysis of covariance (ANCOVA), sex- and age-adjusted values for height by rs17081935 genotype were calculated and expressed as least mean square values (standard error).

Logistic regression was used to calculate odds ratios (ORs) and 95% confidence intervals (CIs) to determine the association between reduced handgrip strength and rs17081935 genotype. We also performed these analyses stratified by atherosclerosis status. Logistic regression was also used to determine the association between reduced handgrip strength and atherosclerosis.

Four adjusted models were used. Model 1 adjusted for sex and age. Model 2 adjusted for sex, age, and cardiovascular risk factors as potential confounders, namely, drinking status (none, often, daily), smoking status (never, former, current), BMI category (< 18.0 kg/m^2^, 18.0–24.9 kg/m^2^, ≥ 25.0 kg/m^2^), hypertension (no, yes), dyslipidemia (no, yes), and diabetes (no, yes). Model 3 adjusted for variables in model 2 plus height (cm). Drinking status, smoking status, BMI category, hypertension, dyslipidemia, and diabetes are known to affect the endothelium, but the condition of the endothelium could not be evaluated based on these factors in this study. Since atherosclerosis evaluated by CIMT and renal function are factors that directly indicate endothelium condition [19, 20] and endothelial repair activity might play a crucial role in maintaining muscle strength among older individuals [21], we also generated a model (model 4) that adjusted only for sex, age, height, and renal function evaluated by GFR category (< 60 mL/min/1.73 m^2^, 60–89 mL/min/1.73m^2^, and ≥ 90 mL/min/1.73 m^2^).

To validate the study population in the present study, the Hosmer–Lemeshow test for goodness of fit were performed. To evaluate the sex-specific correlation between BMI and height, simple correlation analysis was performed.

All statistical analyses were performed using SAS for Windows, version 9.4 (SAS Inc., Cary, NC, USA). Values of *p* < 0.05 were considered statistically significant.

## Results

### Characteristics of the study participants

Regarding rs17081935 genotype, there were 580 CC-homozygotes, 604 heterozygotes, and 190 TT-homozygotes. None of the variables examined were significantly associated with rs17081935 genotype (Table 1). The distribution of this polymorphism was consistent with Hardy–Weinberg equilibrium (*χ*^2^ = 2.601).

**Table 1:** Characteristics of study population by rs17081935 genotype

	rs17081935			*p* value
	C/C	C/T	T/T	
No. of participants	580	604	190	
Men, %	36.7	37.1	32.1	0.439
Age	72.7 ± 7.1	72.8 ± 7.0	72.7 ± 7.0	0.973
Erythrocyte, × 10^4^/μL	445 ± 40	442 ± 42	443 ± 43	0.615
Low BMI (< 18.0 kg/m^2^)	5.5	6.8	5.3	0.584
High BMI (25.0 kg/m^2^ ≤)	23.6	21.7	18.9	0.381
Daily drinker, %	14.7	17.2	18.9	0.289
Non drinker, %	64.3	59.8	57.4	0.132
Current smoker, %	6.4	8.1	10.5	0.158
Former smoker, %	24.5	25.5	21.6	0.550
Hypertension, %	60.2	57.8	60.5	0.649
Dyslipidemia, %	54.8	56.0	54.2	0.884
Diabetes, %	11	11.8	8.4	0.440
CIMT, mm	1.0 ± 0.2	0.9 ± 0.2	1.0 ± 0.2	0.638
Handgrip strength, kg	25.0 ± 8.6	25.0 ± 8.5	25.0 ± 8.5	0.993
Height, cm	155.3 ± 8.2	155.3 ± 8.7	155.9 ± 8.6	0.669
Mild reduced renal function, %	64.8	67.1	66.8	0.702
CKD, %	26.9	25.3	26.3	0.827

*Values* mean ± standard deviation, *BMI* body mass index, *CIMT* carotid intima-media thickness, *CKD* chronic kidney disease

BMI was calculated with data on height, but no significant association between BMI and height was observed in the present study population. The simple correlation coefficient and *p* value for height and BMI were *r* = 0.09 (*p* = 0.058) for men and *r* =  − 0.04 (*p* = 0.199) for women, respectively.

Characteristics of the study population by atherosclerosis status are shown in Supplemental Table 1. Compared to participants without atherosclerosis, participants with atherosclerosis had higher age, CIMT, and height. There was also a higher prevalence of men, former smoking, hypertension, and CKD.

Supplemental Table 2 shows the characteristics of the study participants by handgrip strength. Participants with reduced handgrip strength were significantly older and had a significant higher prevalence of low BMI, non-drinker status, hypertension, and CKD. They had higher CIMT and lower erythrocyte count and height than participants without reduced handgrip strength.

For all logistic regression analyses performed in the present study, goodness of fit for the study population was validated.

### Association between rs17081935 genotype and reduced handgrip strength

Table 2 shows the ORs and 95% CIs for reduced handgrip strength in relation to rs17081935 genotype among all participants. No significant associations between rs17081935 and reduced handgrip strength were observed. No significant association between erythrocyte count and rs17081935 genotype was observed. On the other hand, there was a significantly positive association between a minor allele of rs17081935 and height in the sex- and age-adjusted model.

**Table 2:** Odds ratios (ORs) and 95% confidence intervals (CIs) for reduced handgrips strength in relation to rs17081935 genotypes

	**rs17081935 genotypes**			*p*	Minor allele (T)	[*p*]^b^
	(C/C)	(C/T)	(T/T)			
No. of participants	580	604	190			
No. of case (%)	121 (20.9)	131 (21.7)	33 (17.4)			
Height^a^ (standard error)	155.3 (0.3)	155.3 (0.3)	156.4 (0.4)	0.027		
Erythrocyte, × 10^4^/μL	445 ± 40	442 ± 42	443 ± 43	0.615		
Model 1	Ref	1.05 (0.78, 1.41)	0.78 (0.50, 1.22)	0.465	0.93 (0.76, 1.13)	0.067
Model 2	Ref	1.05 (0.78, 1.41)	0.79 (0.50, 1.24)	0.494	0.93 (0.76, 1.14)	0.078
Model 3	Ref	1.02 (0.75, 1.38)	0.85 (0.53, 1.35)	0.619	0.95 (0.77, 1.17)	0.657
Model 4	Ref	1.02 (0.76, 1.38)	0.85 (0.53, 1.34)	0.620	0.95 (0.77, 1.17)	0.497

^a^: Sex and age adjusted values of height. ^b^: *P* for good for fit test evaluated by the Hosmer–Lemeshow test. Ref: reference. Model 1: adjusted only for sex and age. Model 2: adjusted further for drinking status (non, often, daily), smoking status (never, former, current), BMI status (< 18.0 kg/m^2^, 18.0–24.9 kg/m^2^, and 25 kg/m^2^ ≤), hypertension, dyslipidemia, diabetes. Model 3: further adjusted for height. Model 4: adjusted only for sex, age, height, and renal function (< 60 mL/min/1.73m^2^, 60–89 mL/min/1.73m^2^, 90 ml/min/1.73m^2^ ≤)

### Association between rs17081935 genotype and reduced handgrip strength by atherosclerosis status

Table 3 shows the ORs and 95% CIs for reduced handgrip strength in relation to rs17081935 genotype stratified by atherosclerosis status. No significant associations between a minor allele of rs17081935 and reduced handgrip strength were observed for older participants without atherosclerosis. However, a significant inverse association between a minor allele of rs17081935 and reduced handgrip strength was observed among older participants with atherosclerosis. These associations were unchanged even after further adjustment for known cardiovascular risk factors and height. Table 3 also shows the association between sex- and age-adjusted height and rs17081935 genotype, stratified by atherosclerosis status. We found no significant association between rs17081935 genotype and height for older participants with or without atherosclerosis.

**Table 3:** Odds ratios (ORs) and 95% confidence intervals (CIs) for reduced handgrips strength in relation to rs17081935 genotypes by atherosclerosis

	**rs17081935 genotypes**			*p*	Minor allele (T)	[*p*]^b^
	(C/C)	(C/T)	(T/T)			
**Atherosclerosis ( −)**						
No. of participants	452	475	149			
No. of case (%)	75 (16.6)	101 (21.3)	26 (17.4)			
Height^a^ (standard error)	155.0 (0.3)	155.0 (0.3)	155.9 (0.5)	0.186		
Erythrocyte, × 10^4^/μL	447 ± 40	441 ± 40	441 ± 44	0.098		
Model 1	Ref	1.38 (0.97, 1.95)	1.08 (0.64, 1.81)	0.353	1.12 (0.89, 1.41)	0.057
Model 2	Ref	1.38 (0.97, 1.96)	1.08 (0.64, 1.82)	0.350	1.12 (0.89, 1.41)	0.786
Model 3	Ref	1.34 (0.94, 1.92)	1.11 (0.65, 1.90)	0.343	1.13 (0.86, 1.43)	0.424
Model 4	Ref	1.35 (0.94, 1.93)	1.12 (0.65, 1.91)	0.329	1.13 (0.89, 1.44)	0.402
**Atherosclerosis ( +)**						
No. of participants	128	129	41			
No. of case (%)	46 (35.9)	30 (23.3)	7 (17.1)			
Height^a^ (standard error)	156.2 (0.5)	156.3 (0.5)	158.2 (0.9)	0.116		
Erythrocyte, × 10^4^/μL	438 ± 41	446 ± 46	450 ± 37	0.158		
Model 1	Ref	0.51 (0.29, 0.91)	0.33 (0.13, 0.84)	0.005	0.55 (0.37, 0.83)	0.379
Model 2	Ref	0.47 (0.26, 0.85)	0.32 (0.12, 0.81)	0.003	0.53 (0.34, 0.81)	0.588
Model 3	Ref	0.46 (0.25, 0.84)	0.38 (0.15, 0.99)	0.008	0.55 (0.36, 0.86)	0.712
Model 4	Ref	0.48 (0.27, 0.88)	0.40 (0.16, 1.01)	0.010	0.57 (0.37, 0.88)	0.951

^a^ Sex and age adjusted values of height. ^b^*P* for good for fit test evaluated by the Hosmer–Lemeshow test. Ref: reference. Model 1, adjusted only for sex and age. Model 2, adjusted further for drinking status (non, often, daily), smoking status (never, former, current), BMI status (< 18.0 kg/m^2^, 18.0–24.9 kg/m^2^, and 25 kg/m^2^ ≤), hypertension, dyslipidemia, diabetes. Model 3, further adjusted for height. Model 4, adjusted only for sex, age, height, and renal function (< 60 mL/min/1.73 m^2^, 60–89 mL/min/1.73m^2^, 90 ml/min/1.73m^2^ ≤)

Although there might have been insufficient power, a positive tendency between a minor allele of rs17081935 and erythrocyte count was observed among participants with atherosclerosis.

### Effect of the interaction of atherosclerosis on the association between a minor allele of rs17081935 and reduced handgrip strength

We also found that the status of atherosclerosis had a significant effect on the association between rs17081935 genotype and reduced handgrip strength. The *p* value of the interaction was 0.003 in model 1, 0.003 in model 2, 0.007 in model 3, and 0.007 in model 4 (not shown in Tables).

### Association between atherosclerosis and reduced handgrip strength stratified by rs17081935 genotype

Table 4 shows the association between atherosclerosis and reduced handgrip strength by rs17081935 genotype. Among rs17081935 CC-homozygous participants, those with atherosclerosis had a significantly higher OR for reduced handgrip strength and a significantly lower erythrocyte count than participants without atherosclerosis.

**Table 4:** Odds ratios (ORs) and 95% confidence intervals (CIs) for reduced handgrips strength by atherosclerosis in relation to rs17081935 genotypes

	**Atherosclerosis**		*p*	[*p*]^b^
	( −)	( +)		
**CC-homozygotes**				
No. of participants	452	128		
No. of case (%)	75 (16.6)	46 (35.9)		
Erythrocyte, × 10^4^/μL	447 ± 40	438 ± 41	0.023	
Model 1	Ref	2.13 (1.34, 3.40)	0.002	0.962
Model 2	Ref	2.15 (1.35, 3.45)	0.001	0.839
Model 3	Ref	2.25 (1.39, 3.63)	< 0.001	0.501
Model 4	Ref	2.27 (1.41, 3.67)	< 0.001	0.240
**CT-heterozygotes**				
No. of participants	475	129		
No. of case (%)	101 (21.3)	30 (23.3)		
Erythrocyte, × 10^4^/μL	441 ± 40	446 ± 46	0.238	
Model 1	Ref	0.77 (0.47, 1.27)	0.299	0.529
Model 2	Ref	0.75 (0.46, 1.25)	0.271	0.742
Model 3	Ref	0.79 (0.47, 1.34)	0.379	0.832
Model 4	Ref	0.84 (0.50, 1.41)	0.505	0.734
**TT-homozygotes**				
No. of participants	149	41		
No. of case (%)	26 (17.4)	7 (17.1)		
Erythrocyte, × 10^4^/μL	441 ± 44	450 ± 37	0.271	
Model 1	Ref	0.60 (0.22, 1.63)	0.320	0.433
Model 2	Ref	0.55 (0.19, 1.58)	0.269	0.299
Model 3	Ref	0.74 (0.26, 2.16)	0.586	0.321
Model 4	Ref	0.77 (0.27, 2.15)	0.614	0.090

^b^: *P* for good for fit test evaluated by the Hosmer–Lemeshow test. Ref: reference. Model 1, adjusted only for sex and age. Model 2, adjusted further for drinking status (non, often, daily), smoking status (never, former, current), BMI status (< 18.0 kg/m^2^, 18.0–24.9 kg/m^2^, and 25 kg/m^2^ ≤), hypertension, dyslipidemia, diabetes. Model 3, further adjusted for height. Model 4, adjusted only for sex, age, height, and renal function (< 60 mL/min/1.73m^2^, 60–89 mL/min/1.73m^2^, 90 ml/min/1.73 m^2^ ≤)

For both rs17081935 CT-heterozygotes and CC-homozygotes, participants with atherosclerosis had a lower OR for reduced handgrip strength and a higher erythrocyte count than participants without atherosclerosis, even though these associations were not statistically significant.

### Sensitivity analyses

Sex-specific analyses were performed with an age-adjusted model. Essentially, the same associations were observed between men and women. The ORs and 95% CIs for reduced handgrip strength and a minor allele of rs17081935 were 1.24 (0.81, 1.89) for men without atherosclerosis, 0.65 (0.37, 1.17) for men with atherosclerosis, and 1.07 (0.81, 1.41) for women without atherosclerosis, and 0.45 (0.21, 0.82) for women with atherosclerosis. Among CC-homozygotes, age-adjusted ORs and 95% CIs for reduced handgrip strength and atherosclerosis were 2.72 (1.33, 5.56) for men and 1.28 (0.72, 2.26) for women, respectively.

We also re-performed the main analyses using two different definitions of reduced handgrip strength. The first definition for reduced handgrip strength is being in the lowest quartile of handgrip strength (< 27.6 kg for men and < 17.1 kg for women). The second definition for reduced handgrip strength was based on the 2019 Asian Working Group for Sarcopenia (AWGS) criteria for diagnosing sarcopenia (< 28.0 kg for men and < 18.0 kg for women). We obtained essentially the same results. In the fully adjusted model, among older participants without atherosclerosis, the ORs and 95% CIs for reduced handgrip strength based on the first and second definitions for a minor allele of rs17081935 were 1.09 (0.87, 1.36) and 1.02 (0.82, 1.28), respectively. Among participants with atherosclerosis, the corresponding values were 0.63 (0.42, 0.93) and 0.63 (0.43, 0.94). Among CC-homozygous participants, atherosclerosis was significantly positively associated with reduced handgrip strength based on either definition. The fully adjusted OR (95% CI) was 2.04 (1.28, 3.25) for the first definition and 1.84 (1.16, 2.92) for the second definition.

## Discussion

The major findings of the present study are that a minor allele of the tall stature-related SNP rs17081935 is inversely associated with reduced handgrip strength among older Japanese individuals with atherosclerosis but not among those without atherosclerosis, independent of known cardiovascular risk factors and height. Among CC-homozygotes for the rs17081935 SNP, participants with atherosclerosis had significantly lower erythrocyte counts than participants without atherosclerosis. Among minor allele carriers of rs17081935, participants with atherosclerosis had non-significantly higher erythrocyte counts than those without atherosclerosis.

Previously, our study with 537 older Japanese aged 60–89 years showed that a minor allele of BRAP (BRCA1 associated protein) (rs3782886) is significantly positively associated with short stature and reduced muscle strength evaluated based on tongue pressure [10]. In the present study, we found evidence that the tall stature-related SNP rs17081935 is independently inversely associated with reduced muscle strength evaluated based on handgrip strength in older participants with atherosclerosis but not among those without atherosclerosis. These associations remained even after adjusting for known cardiovascular risk factors and height.

However, the mechanism that underlies the present results has not yet been clarified. Although the SNP rs17081935 is known to be associated with height [4, 5], height is not the main explanation for the present results because the significant inverse association between a minor allele of rs17081935 and reduced muscle strength persisted even after adjusting for height among older participants with atherosclerosis. There were also no significant associations between the SNP rs17081935 and height among those participants.

Mitochondrial metabolism might play an important role since the rs17081935 (REST-NOA1) SNP encodes nitric oxide associated 1 (NOA1) protein [4, 5]. NOA1 is transcriptionally regulated in an oxygen-sensitive manner and supports mitochondrial oxidative phosphorylation, which regulates mitochondrial metabolism [22]. Mitochondrial energy production declines with aging in muscle cells. This age-related decline impairs oxidative phosphorylation, which results in reduced muscle strength [23]. In the present study, the inverse association between a minor allele of rs17081935 and reduced handgrip strength was limited to older participants with atherosclerosis (Table 3). Oxidative stress, which is known to be associated with atherosclerosis [7], also causes mitochondrial dysfunction [2]. Therefore, increased levels of oxidative stress induce both age-related reduced muscle strength [24] and atherosclerosis [7].

The rs17081935 SNP has a beneficial influence on maintaining muscle strength in older participants with atherosclerosis, suggesting that this beneficial influence is supportive; this genetic characteristic may help prevent muscle strength loss only among older with increased levels of oxidative stress.

Since oxidative stress reduces erythrocyte survival and induces eryptosis, the number of erythrocytes in peripheral blood decreases [8]. In the present study, among CC-homozygotes, participants with atherosclerosis had a significant higher OR for reduced handgrip strength and lower erythrocyte count than participants without atherosclerosis (Table 4). Therefore, these results indicate that, among participants without the influence of the minor allele of rs17081935, those with atherosclerosis had significantly lower erythrocyte counts and significantly higher ORs for reduced handgrip strength due to the presence of increased oxidative stress.

We found non-statistically significant but interesting associations for CT-heterozygotes and TT-homozygotes: participants with atherosclerosis had a lower ORs for reduced handgrip strength and higher erythrocyte count than participants without atherosclerosis (Table 4). These results indicate that, under the influence of the minor allele of rs17081935, a reduction in erythrocyte count related to increased oxidative stress (atherosclerosis) could not be observed. Furthermore, minor allele carriers of rs17081935 might have a lower rate of erythrocyte reduction related to atherosclerosis.

Since mitochondrial metabolism could influence cell survival [25], those associations could be explained by the beneficial effect of activating mitochondrial metabolism in CT-heterozygotes and TT-homozygotes, which might reduce the influence of oxidative stress on erythrocyte count.

Among participants with atherosclerosis, there was a non-statistically significant positive tendency between a minor allele (T) of rs17081935 and erythrocyte count (Table 3). Among such participants, a significant inverse association between a minor allele (T) of rs17081935 and reduced handgrip strength was observed (Table 3).

Since repeated exercise reportedly causes muscle adaptations to hypoxia such as the development of additional capillaries [9], angiogenesis related to maintaining muscle strength might also reduce oxidative stress, which prevents erythrocyte reduction in peripheral blood [8]. Therefore, reduced handgrip strength was associated with lower erythrocyte count in the present study (Supplemental Table 2).

These results also support the hypothesis that the beneficial influence of carrier of a miner allele (T) of rs17081935 on maintaining muscle strength could be enhanced by oxidative stress related to atherosclerosis.

This is the first study that reports a tall stature-related genetic factor having a beneficial influence on muscle strength maintenance among older individuals independent of height. The present findings might help clarify the mechanisms responsible for muscle strength loss among older individuals.

The potential limitations of the present study warrant consideration. NOA1 and oxidative stress might have substantially affected the present results. However, we have no information about NOA1 and oxidative stress. To clarify the background mechanisms leading to the present results, further investigation with data on NOA1 and oxidative stress is necessary. Unknown confounding factors that influence mitochondrial metabolism might have affected the present results. Further study with data such as thyroid hormone levels [26], other genetic factors, and mental distress are necessary. Even though reduced erythrocyte count might act as an indicator of higher levels of oxidative stress [8], among participants with atherosclerosis, no significant association between a minor allele (T) of rs17081935 and erythrocyte count was observed. However, a non-significant positive association was observed between a minor allele (T) of rs17081935 and erythrocyte count, which supports the mechanisms we propose.

## Conclusions

In conclusion, a minor allele of the rs17081935 SNP was significantly inversely associated with reduced handgrip strength among older individuals with atherosclerosis but not those without atherosclerosis. Among CC-homozygotes for the rs17081935 SNP, participants with atherosclerosis had significantly lower erythrocyte counts than participants without atherosclerosis. Among minor allele carriers of rs17081935, participants with atherosclerosis had non-significantly higher erythrocyte counts than those without atherosclerosis. These results can help clarify some of the mechanisms underlying age-related muscle strength loss.

## Supplementary Information

**Additional file 1: Supplemental Table 1**. Characteristics of study population by status of atherosclerosis.

**Additional file 2: Supplemental Table 2. **Characteristics of study population by status of reduced handgrip strength.

## Data Availability

The datasets generated during and/or analyzed during the current study are not publicly available due to ethical consideration but are available from the corresponding author on reasonable request.

## Abbreviations

SNP: Single nucleotide polymorphism
OR: Odd ratio
CI: Confidence interval
SD: Standard deviation
BMI: Body mass index
SBP: Systolic blood pressure
DBP: Diastolic blood pressure
LDLc: Low-density lipoprotein-cholesterol
HDLc: High-density lipoprotein-cholesterol
TG: Triglycerides
HbA1c: Glycated hemoglobin
GFR: Glomerular filtration rate
PCR: Polymerase chain reaction
FRET: Fluorescence resonance energy transfer
CIMT: Carotid intima-media thickness
ANCOVA: Analysis of covariance
ORs: Odd ratios
CIs: Confidence intervals
AWGS: Asian Working Group for Sarcopenia
BRAP: BRCA1 associated protein
NOA1: Nitric oxide associated 1
