# Supplementary material for: Association between height-related polymorphism rs17081935 and reduced handgrip strength in relation to status of atherosclerosis: a cross-sectional study
Source: Environ Health Prev Med. 2021 Aug 26;26:83. doi: 10.1186/s12199-021-01000-9 (PMC8393436; doi:10.1186/s12199-021-01000-9)
Supplement: Supplementary file 1 — Additional file 1: Supplemental Table 1. Characteristics of study population by status of atherosclerosis. [file 12199_2021_1000_MOESM1_ESM.docx]

| **Supplemental Table 1.** | | **Characteristics of study population by status of atherosclerosis** | | | |
| --- | --- | --- | --- | --- | --- |
|  |  |  | Atherosclerosis | | p value |
|  |  |  | (-) | (+) |  |
|  | No. of participants | | 1076 | 473 |  |
|  | Men, % | | 33.2 | 47.3 | <0.001 |
|  | Age | | 72.0 ± 7.0 | 75.7 ± 6.5 | <0.001 |
|  | Erythrocyte, ×10^4^/μL | | 444 ± 41 | 443 ± 43 | 0.824 |
|  | Low BMI (<18.0kg/m^2^) | | 6.7 | 3.7 | 0.054 |
|  | High BMI (25.0kg/m^2^≤) | | 21.9 | 22.8 | 0.745 |
|  | Daily drinker, % | | 15.8 | 18.5 | 0.273 |
|  | Non drinker, % | | 61.9 | 59.4 | 0.433 |
|  | Current smoker, % | | 7.5 | 8.4 | 0.622 |
|  | Former smoker, % | | 22.1 | 33.2 | <0.001 |
|  | Hypertension, % | | 57.4 | 65.4 | 0.013 |
|  | Dyslipidemia, % | | 54.1 | 59.4 | 0.103 |
|  | Diabetes, % | | 10.2 | 13.8 | 0.084 |
|  | CIMT, mm | | 0.9 ± 0.1 | 1.3 ± 0.2 | <0.001 |
|  | Handgrip strength, kg | | 25.0 ± 8.4 | 25.1 ± 9.0 | 0.790 |
|  | Height, cm | | 155.1 ± 8.3 | 156.5 ± 9.2 | 0.013 |
|  | Mild reduced renal function, % | | 68.3 | 58.1 | <0.001 |
|  | CKD, % | | 24.1 | 33.6 | 0.001 |
|  | Values: mean ± standard deviation. CIMT: carotid intima-media thickness. CKD: chronic kidney disease. | | | | |
|  |  |  |  |  |  |
