# Supplementary material for: Association between height-related polymorphism rs17081935 and reduced handgrip strength in relation to status of atherosclerosis: a cross-sectional study
Source: Environ Health Prev Med. 2021 Aug 26;26:83. doi: 10.1186/s12199-021-01000-9 (PMC8393436; doi:10.1186/s12199-021-01000-9)
Supplement: Supplementary file 2 — Additional file 2: Supplemental Table 2. Characteristics of study population by status of reduced handgrip strength. [file 12199_2021_1000_MOESM2_ESM.docx]

| **Supplemental Table 2.** | | **Characteristics of study population by status of reduced handgrip strength** | | | |
| --- | --- | --- | --- | --- | --- |
|  |  |  |  |  |  |
|  |  |  | Reduced handgrip strength | | p value |
|  |  |  | (-) | (+) |  |
|  | No. of participants | | 1089 | 285 |  |
|  | Men, % | | 36.7 | 34.4 | 0.464 |
|  | Age | | 71.6 ± 6.7 | 77.1 ± 6.9 | <0.001 |
|  | Erythrocyte, ×10^4^/μL | | 446 ± 40 | 433 ± 44 | <0.001 |
|  | Low BMI (<18.0kg/m^2^) | | 5.1 | 9.8 | 0.003 |
|  | High BMI (25.0kg/m^2^≤) | | 21.9 | 29.8 | 0.756 |
|  | Daily drinker, % | | 17.6 | 11.6 | 0.014 |
|  | Non drinker, % | | 59.4 | 68.8 | 0.004 |
|  | Current smoker, % | | 8.0 | 6.7 | 0.457 |
|  | Former smoker, % | | 25.5 | 20.7 | 0.092 |
|  | Hypertension, % | | 57.2 | 66.7 | 0.004 |
|  | Dyslipidemia, % | | 55.8 | 53.0 | 0.390 |
|  | Diabetes, % | | 10.4 | 13.3 | 0.156 |
|  | CIMT, mm | | 0.9 ± 0.2 | 1.0 ± 0.2 | <0.001 |
|  | Handgrip strength, kg | | 27.3 ± 7.8 | 16.3 ± 4.8 | <0.001 |
|  | Height, cm | | 156.4 ± 8.2 | 151.8 ± 8.5 | <0.001 |
|  | Mild reduced renal function, % | | 68.6 | 56.5 | <0.001 |
|  | CKD, % | | 23.9 | 34.7 | <0.001 |
|  | Values: mean ± standard deviation. CIMT: carotid intima-media thickness. CKD: chronic kidney disease. | | | | |
|  |  |  |  |  |  |
